# Evaluation of Hypovirus Infection on the Vesicular Protein Expression Pattern of *Cryphonectria parasitica* by TMT-Based Proteomics Analysis

**DOI:** 10.3390/biology14091123

**Published:** 2025-08-25

**Authors:** Zishan Zeng, Nanxin Lin, Tao Lu, Jian Xu, Zheng Zhang, Fang Wang, Jinzi Wang

**Affiliations:** 1 Guangxi Key Laboratory for Polysaccharide Materials and Modifications, School of Marine Sciences and Biotechnology, Guangxi Minzu University, Nanning 530008, China; 202312141201359@stu.gxmzu.edu.cn (Z.Z.); nanxinlingxmzu@163.com (N.L.);; 2 Guangxi Aquatic Animal Husbandry School, Nanning 530021, China

**Keywords:** *Cryphonectria parasitica*, hypovirus, differential proteomic analysis, vesicle-mediated intracellular transport

## Abstract

Hypovirus infection is known to reduce the pathogenicity of *Cryphonectria parasitica*, the causative agent of chestnut blight. Isoforms derived from a viral protein p48 have been discovered in host mitochondria and vesicles, which may contribute to virulence attenuation, as reported in earlier work using two-dimensional electrophoresis (2-DE). In this study, a total of 1739 fungal proteins were identified in fungal vesicles through Tandem Mass Tag (TMT)-based quantitative proteomics. The infection of CHV1-EP713 was associated with 75 up-regulated and 201 down-regulated proteins, predominantly involved in vesicular transport process and related cellular functions, including protein folding, membrane fusion, retrograde transport, autophagy, and ER stress responses. The down-regulation of calnexin, COPI, ArfGAP, importin-β, and Atg8 is consistent with impairments in protein folding, retrograde transport, and autophagy. Meanwhile, the up-regulation of clathrin, dynamin, Vps10p, HSP70, and t-SNAREs indicated enhanced trafficking to vacuoles and increased stress response activity. Overall, our findings indicate that hypoviral infection is associated with extensive alterations in the vesicular transport system of *C. parasitica*, likely mediated through changes in the abundance of multiple key protein regulators. These alterations may underlie attenuation of virulence by impacting crucial cellular processes.

## Simple Summary

The subject of this study is the alterations in the protein composition of vesicles within *Cryphonectria parasitica* infected by a hypovirus. The experimental results revealed that viral infection modulates the vesicular transport system of vesicles in the host. These alterations may facilitate viral trafficking and replication within the host. Meanwhile the presence of viral proteins in fungal vesicles was observed using tandem mass tag technology, providing insights into the potential biological control strategies for plant fungal diseases.

## 1. Introduction

*Cryphonectria parasitica* is native to East Asia and spreads to other continents via infected chestnut plants. This pathogen causes chestnut blight, a devastating disease affecting American and European chestnut trees. *C. parasitica* induces necrotic lesions on the bark of stems and branches of susceptible host trees, ultimately leading to wilting of the plant parts distal to the infection site. These lesions are characterized by abundant fungal mycelium and spores, and below the lesion, the tree may respond by producing epicormic shoots [1].

Hypoviruses are positive-strand RNA viruses that are located in the cytoplasm of the fungal host. Hypoviruses uniquely attenuate fungal virulence, offering a promising avenue for developing innovative biological control strategies against plant pathogens [2]. The genus *Hypovirus* currently comprises four well-characterized viruses: Cryphonectria hypovirus 1 (CHV-1), CHV-2, CHV-3 and CHV-4 [3,4]. CHV-1 induces a hypovirulent phenotype in the chestnut blight fungus *C. parasitica* by infecting it [5]. CHV-2 and CHV-3 also induce a hypovirulent phenotype in *C. parasitica*, whereas CHV-4 causes no significant symptoms in the fungal host [3]. The hypovirulent phenotype is influenced by both horizontal and vertical transmission of the hypovirus. The virus is transmitted only through asexual spores and not through sexual ascospores [6]. Hypovirus infection also results in marked changes in multiple traits of the fungal host, such as altered colony pigmentation, suppression of asexual spore production, female sterility, and modifications in gene expression patterns [2].

Transcriptional analysis has revealed extensive alterations in gene expression in the hosts infected by hypoviruses [7]. Comparative proteomics is an effective tool for investigating virus–host interactions [8]. Research on the total protein content of *C. parasitica* was first reported in 1987, revealing that viral infection reduces the accumulation of virulence-associated proteins and significantly alters fungal virulence [9]. Since then, proteomic studies have yielded substantial insights into fungal virulence regulation and virus–host interactions. Studies have revealed significant changes in protein expression under hypovirus regulation and environmental stress (e.g., tannic acid) [10]. Hypovirus infection is associated with disruption of secretion pathways, notably inhibiting the secretion rate of cryparin, a key hydrophobin, and causing its intracellular accumulation. This disruption suggests possible the virus-mediated regulation of the trans-Golgi network (TGN) secretion pathway [11,12].

Our previous research had preliminarily compared the differences in vesicle proteome between hypovirus-free *C. parasitica* strain EP155 and its isogenic strain EP713 (hypovirus CHV1-EP713-infected EP155) by two-dimensional electrophoresis (2-DE) coupled with mass spectrometry. It is found that 33 protein spots expressed differentially were identified on the 2-DE gels. Furthermore, a virus-encoded protein p48 was found to have four forms with different molecular mass in vesicles from the virus-infected strain via an undefined mechanism [13]. Due to the limited resolution and sensitivity of 2-DE, these findings only allowed a rough inference that CHV1 infection alters the host vesicular system.

In parallel, Chun et al. [14] performed a transcriptome-wide RNA sequencing (RNA-seq) analysis, revealing large-scale gene expression changes upon CHV1 infection, particularly in metabolic and transport processes. Both their study and our earlier proteomic analysis consistently identified up-regulation of HSP70, underscoring its potential as a core component of the CHV1–host interaction. However, transcriptomic approaches are unable to directly assess protein abundance, subcellular localization, or post-translational regulation, and our prior work revealed substantial discrepancies between transcript and protein levels.

To overcome these limitations and expand the detectable vesicle proteome, the present study employs tandem mass tag (TMT)-based quantitative proteomics integrated with parallel reaction monitoring (PRM) validation. This approach not only detected over 1700 vesicle-associated proteins, far exceeding the coverage of 2-DE, but also quantified low-abundance proteins and vesicle-specific changes inaccessible to transcriptomics. This integrated strategy markedly improves proteome coverage, quantitative accuracy, and subcellular resolution, thereby enabling a more comprehensive exploration of the subtle alterations in the vesicular transport system of *C. parasitica* during hypovirus infection.

## 2. Materials and Methods

### 2.1. Fungal Strains and Culture Conditions

The wild-type *C. parasitica* strain EP155 (ATCC 38755) and its isogenic strain EP155/CHV1-EP713, harboring hypovirus CHV1-EP713 acquired via horizontal transmission, were used in this study. For simplicity, the strain EP155/CHV1-EP713 is hereafter referred to as EP713 throughout the manuscript. Both strains were routinely cultured on potato dextrose agar (PDA) at 25 °C under a 12 h light/dark cycle.

### 2.2. Vesicle Sample Preparation and Analysis

Fungal mycelia were finely pulverized under liquid nitrogen and mixed with 0.1 M sodium acetate buffer containing 0.07% β-mercaptoethanol) at a mass-to-volume ratio of 1:3 (g·mL^−1^). The mixture was gently stirred for 2 h at 4 °C, followed by centrifugation at 6500 r·min^−1^ for 30 min at 4 °C. The resulting pellet was resuspended in the same buffer and centrifuged again under identical conditions to obtain a second supernatant, which was retained. The two supernatants were combined and centrifuged once more at 7500 r·min^−1^ for 30 min at 4 °C. The resulting supernatant was ultracentrifuged at 50,000 r·min^−1^ for 90 min. The remaining pellet was dissolved in TMD buffer (50 mM Tris, pH 7.5, 10 mM MgCl_2_, 5 mM DTT), and the supernatant was discarded. The solution was concentrated to a final volume of 50–100 µL at 4 °C using a Pierce protein concentrator (8000 r·min^−1^) and stored at −80 °C for in further studies.

A total of 400 μL vesicle sample was mixed with 200 μL membrane protein lysis buffer containing 5 M urea, 2 M thiourea, 2% CHAPS, 2% SB3-10, 40 mM Tris, 5 mM β-mercaptoethanol, and 0.17% protease inhibitor. After thorough mixing, 3 L of cold acetone containing 0.07% β-mercaptoethanol and 10% trichloroacetic acid was added, and the mixture was incubated at −20 °C for 30 min to precipitate proteins. The sample was centrifuged at 13,000 r·min^−1^ for 30 min at 4 °C, and the pellet was washed three times with cold acetone that containing 0.07% β-mercaptoethanol. After air drying, the pellet was dissolved in 200 μL membrane protein lysis buffer. Protein concentration was determined by the Bradford method using Coomassie Brilliant Blue G-250 as the dye. Proteins were then analyzed by SDS-PAGE (12.5%).

For scanning electron microscopy (SEM), an appropriate volume of purified vesicle samples was applied to glass coverslips, air-dried, and fixed sequentially in 30%, 50%, 70%, 90%, and 100% ethanol, followed by fixation in 2.5% glutaraldehyde. Coverslip sections were cut and mounted on aluminum stubs with conductive adhesive, coated with platinum, and observed under a scanning electron microscope (Zeiss Supra55, Baden-Württemberg, Germany).

### 2.3. Protein Extraction and Digestion

Proteins were extracted using SDT buffer (4%SDS, 100 mM Tris-HCl, 1 mM DTT, pH 7.6). Protein concentration was determined using the BCA Protein Assay Kit (Bio-Rad, Hercules, CA, USA). Protein digestion was performed using the filter-aided sample preparation (FASP) method as described by Wiśniewski et al. [15].

For FASP digestion, 200 μg of protein from each samples was mixed with 30 μL SDT buffer (4% SDS, 100 mM DTT, 150 mM Tris-HCl pH 8.0). Detergent, DTT, and other low-molecular-weight components were removed by repeated ultrafiltration (Microcon units, 10 kD) with UA buffer (8 M Urea, 150 mM Tris-HCl pH 8.0). Iodoacetamide (100 μL, 100 mM IAA in UA buffer) was then added to alkylate cysteine residues and samples were incubated for 30 min in the dark. Filters were washed three times with 100 μL UA buffer and twice with 100 μL 25 mM ammonium bicarbonate. Proteins were digested with 4 μg sequencing-grade trypsin (Promega, Madison, WI, USA) in 40 μL 25 mM ammonium bicarbonate overnight at 37 °C. Resulting peptides were collected by centrifugation.

Peptides were desalted using C18 solid-phase extraction cartridges (Empore™ SPE Cartridges C18, standard density, bed I.D. 7 mm, volume 3 mL, Sigma-Aldrich, Burlington, VT, USA), dried in a vacuum centrifuge, and reconstituted in 40 µL of 0.1% (*v*/*v*) formic acid. Peptide concentrations were determined by measuring UV absorbance at 280 nm using an extinction coefficient of 1.1 of 0.1% (g/L) solution, calculated based on the frequency of tryptophan and tyrosine residues in vertebrate proteins.

### 2.4. Strong Cation Exchange (SCX) Fractionation

A total of 100 μg peptides from each sample was labeled using TMT reagent according to the manufacturer’s instructions (Thermo Scientific, Waltham, MA, USA). Labeled peptides were fractionated by SCX chromatography using the AKTA Purifier system (GE Healthcare, Waukesha, WI, USA). The dried peptide mixture was reconstituted and acidified with buffer A (10 mM KH_2_PO_4_ in 25% of ACN, pH 3.0) and loaded onto a PolySULFOETHYL 4.6 × 100 mm column (5 µm, 200 Å, PolyLC, Waltham, MA, USA). The peptides were eluted at a flow rate of 1 mL/min with the following gradient of 0% buffer B (500 mM KCl, 10 mM KH_2_PO_4_ in 25% of ACN, pH 3.0): 0% for 0–25 min, 0–10% during 25–32 min, 10–20% during 32–42 min, 20–45% during 42–47 min, 45–100% during 47–52 min, 100% during 52–60 min, followed by re-equilibration to 0% buffer B after 60 min. The elution was monitored by absorbance at 214 nm, and fractions were collected every 1 min. The collected fractions were desalted on C18 cartridges (Empore™ SPE Cartridges C18, standard density, bed I.D. 7 mm, volume 3 mL, Sigma) and concentrated by vacuum centrifugation.

### 2.5. High pH Reversed-Phase Fractionation

Labeled peptides were fractionated using the high-pH Reversed-Phase Peptide Fractionation Kit (Thermo Scientific). The dried peptide mixture was reconstituted and acidified with 0.1% TFA solution and loaded to the equilibrated high-pH reversed-phase fractionation spin column. Peptides were bound to the hydrophobic resin under aqueous conditions and desalted by washing the column with water at low-speed centrifugation. A step gradient of increasing acetonitrile concentrations in a volatile high-pH elution solution was applied to the columns to elute bound peptides into 10 fractions, collected by centrifugation. The collected fractions were desalted on C18 Cartridges (Empore™ SPE Cartridges C18, standard density), bed I.D. 7 mm, volume 3 mL, Sigma) and concentrated by vacuum centrifugation.

### 2.6. LC-MS/MS Analysis

LC-MS/MS analysis was performed on a Q Exactive mass spectrometer (Thermo Scientific) coupled to Easy nLC (Proxeon Biosystems, now Thermo Fisher Scientific) for 60 or 90 min runs. Peptides were loaded onto a reverse-phase trap column (Thermo Scientific Acclaim PepMap100, 100 μm × 2 cm, nanoViper C18) connected to a C18 reversed-phase analytical column (Thermo Scientific Easy Column, 10 cm long, 75 μm inner diameter, 3 μm resin) in buffer A (0.1% formic acid) and separated using a linear gradient of buffer B (84% acetonitrile with 0.1% formic acid) at a flow rate of 300 nL/min controlled by IntelliFlow technology. The mass spectrometer was operated in positive-ion mode.

MS data were acquired using a data-dependent Top10 method dynamically selecting the most abundant precursor ions from the survey scan (*m*/*z* 300–1800) for higher-energy collisional dissociation (HCD) fragmentation. The automatic gain control (AGC) target was set to 3 × 10^6^, with a maximum injection time of 10 ms. The dynamic exclusion duration was 40 s. Survey scans were acquired at a resolution of 70,000 at *m*/*z* 200, and HCD spectra were acquired at a resolution of 17,500 at *m*/*z* 200, with an isolation width of 2 *m*/*z*. The normalized collision energy was 30 eV and the underfill ratio (minimum percentage of the target value likely to be reached at maximum fill time) was set to 0.1%. Peptide recognition mode was enabled.

### 2.7. Identification and Quantitation of Proteins

The MS raw data for each sample were searched using the MASCOT engine (Matrix Science, London, UK; version 2.2) embedded into Proteome Discoverer 1.4 software for identification and quantitation analysis.

### 2.8. Bioinformatic Analysis

Bioinformatics analysis included hierarchical clustering using Cluster 3.0 and Java Treeview 5.0, with Euclidean distance and average linkage algorithms to measure similarity and cluster data, visualized as dendrograms and heat maps. Gene ontology (GO) terms were annotated using NCBI BLAST+ and Blast2GO 2.16, while protein domain annotations were obtained using InterProScan with the Pfam database. Data visualization was performed in R.

For orthology assignment and KEGG pathway mapping, blasting protein sequences were compared against the KEGG database using BLAST. Enrichment analysis was performed using Fisher’s exact test with Benjamini–Hochberg correction for multiple testing, and pathways or categories with *p* < 0.05 were considered significant.

## 3. Results and Discussion

### 3.1. Differential Proteomic Analysis of Fungal Vesicle

**Figure 1:**
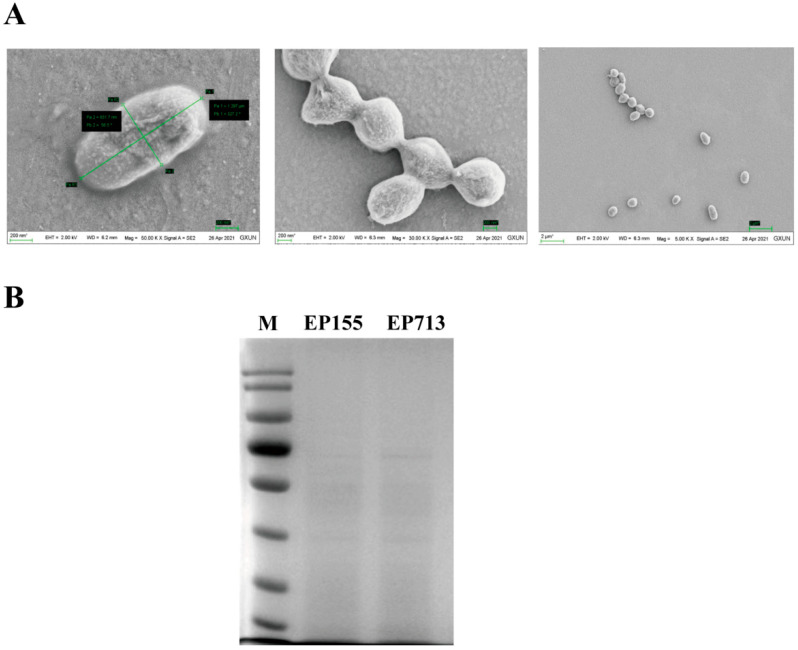
An overview of the differential protein expression is provided, comparing the vesicular proteins of EP155 and EP713. (**A**) Electron microscope observation of *C. parasitica* vesicle sample; (**B**) Electrophoresis analysis of *C. parasitica* vesicle protein samples.

Western blot analysis was performed as previously described (Figure S1) [13]. Furthermore, morphological characterization by scanning electron microscopy revealed extracted fungal vesicles with intact and uniform spherical structures (400–800 nm), as well as a clean background (Figure 1A). Protein electrophoresis analysis demonstrated clear, evenly distributed protein bands with moderate abundance, confirming the purity and integrity of the extracellular vesicle preparation (Figure 1B).

**Figure 2:**
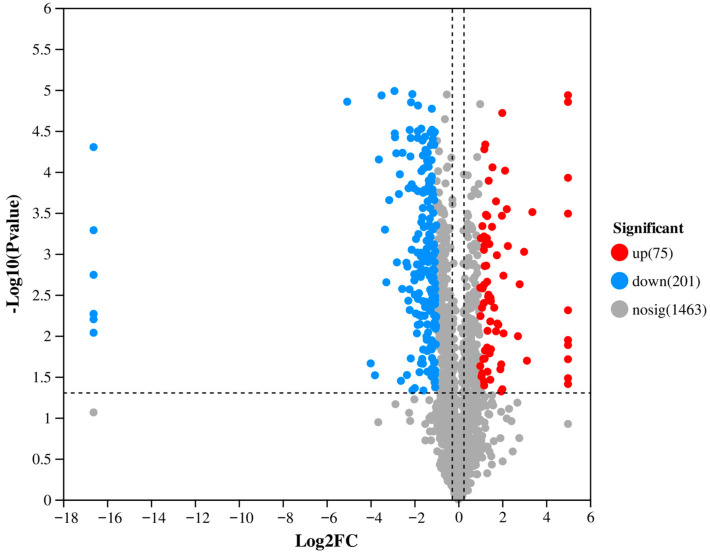
Volcano plot showing the distribution of differentially expressed proteins between EP713 and EP155.

Based on the TMT quantitative proteomics method, a total of 1739 proteins were effectively identified from fungal vesicle samples (Table S1). Compared with the data of wild strain EP155, the hypovirus-infected vesicle proteomic data showed a significant up-regulation of 75 proteins (FC > 2.0, *p* < 0.05) and down-regulation of 201 proteins (FC < 0.5, *p* < 0.05) (Figure 2, Table S3). In order to verify the proteomic results, 25 target peptides of 14 target proteins were randomly selected for PRM analysis (Table S2). The PRM results were consistent with the TMT-based quantification for 10 proteins. Additionally, we identified viral peptide corresponding to proteins encoded by both ORFA and ORFB. Notably, from ORFA, we detected peptides belonging to the final processed proteins p29 and p40 (derived from the p69 polyprotein precursor) [16,17], while from ORPFB, we identified p48, which is associated with CHV1 pathogenicity (Table 1) [18].

**Figure 3:**
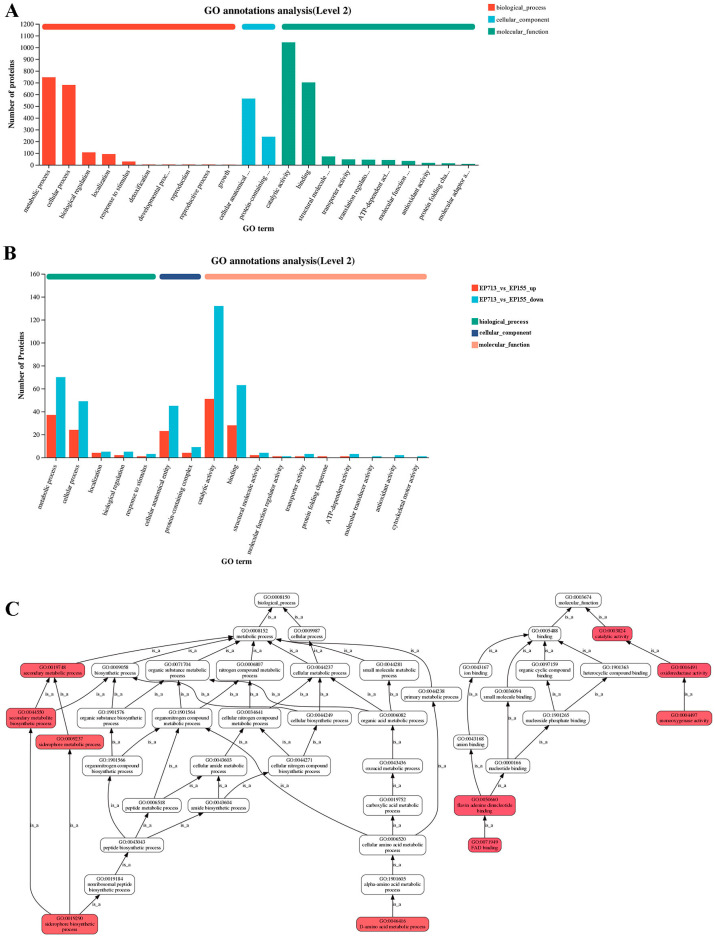
The analysis focuses on protein distribution and enrichment based on GO annotation results. (**A**) The Level 2 GO annotation bar chart shows the functional distribution of vesicle proteins in *C. parasitica*; (**B**) The expression of EP713 changed significantly in key GO categories such as metabolic process, catalytic activity, and binding function; (**C**) Hierarchical GO enrichment analysis of strain EP713 showing enrichment in metabolic processes and catalytic activity, supporting the hypothesis of viral modulation of host metabolic functions and stress responses.

Gene Ontology (GO) annotation analysis revealed that most of the regulated proteins were related to catalytic activity and binding functions (Figure 3A). Further analysis indicated marked changes in expression of EP713 in key GO categories such as metabolic processes, catalytic activity, and binding functions (Figure 3B). Hierarchical GO analysis further suggested a broad effect of hypoviral infection on multiple metabolic pathways (Figure 3C). Significant enrichment differences between EP713 and EP155 were observed in terms related to FAD binding and D-amino acid metabolism, implying that hypovirus infection could alter host metabolism and energy utilization. Moreover, secondary metabolic process and metabolite biosynthesis were significantly enriched, which may be associated with host response to viral infection stress.

**Figure 4:**
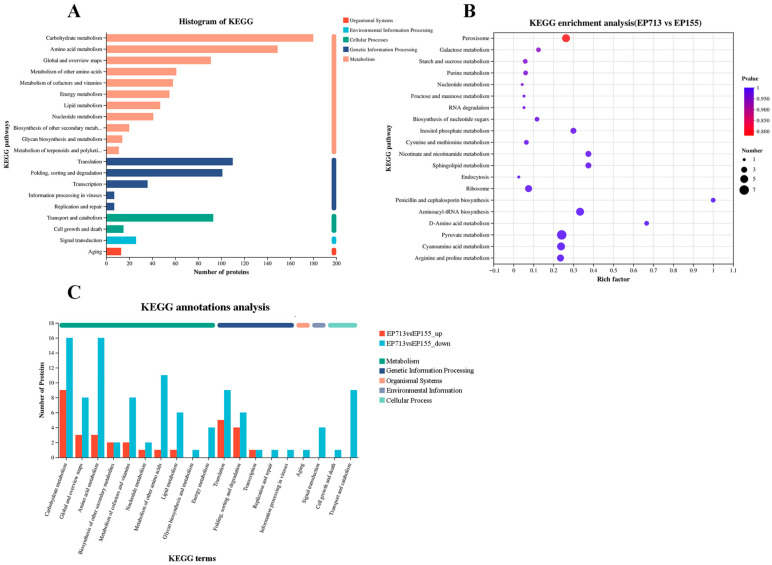
KEGG Annotation and Enrichment Analysis. (**A**) The histogram of KEGG annotations shows the distribution of proteins across different pathways, with a significant number of proteins engaged in amino acid, carbohydrate, energy, and nucleotide metabolism, as well as in processes that maintain protein homeostasis, signal transduction, and genetic information processing; (**B**) KEGG enrichment analysis reveals enrichment trends in metabolic pathways in the EP713 strain, such as peroxisome, galactose metabolism, and starch and sucrose metabolism; (**C**) The distribution of differentially expressed proteins shows significant down-regulation in key pathways, including metabolism, protein synthesis, cellular signaling, and genetic information processing, in EP713.

KEGG pathway analysis indicated enrichment of metabolic pathways, including peroxisome, galactose metabolism, and starch and sucrose metabolism, upon hypoviral infection (Figure 4). However, no pathways remained statistically significant after multiple testing correction, likely due to the limited number of differentially expressed proteins. The adjusted *p*-values for all pathways have been provided in the Supplementary Materials (Table S4).

### 3.2. Potential Impact of Hypovirus on Vesicle Trafficking and ER Homeostasis

Calnexin, an essential endoplasmic reticulum (ER) chaperone, interacts with COPI through its C-terminal di-lysine motif, ensuring its retention in the ER to support protein folding and quality control [19]. In EP713, calnexin is down-regulated, which may compromise ER homeostasis and protein quality control, potentially triggering ER stress. These disturbances could affect processes such as cell wall remodeling, membrane composition, and energy metabolism, ultimately contributing to the observed hypovirulence [20].

The coatomer complex (COPI), which mediates retrograde transport from the Golgi to the ER, is regulated by ArfGAP through its control of Arf protein activity [21]. In EP713, both COPI and ArfGAP are down-regulated, which may impair vesicle formation and disrupt retrograde trafficking of ER-resident proteins [22,23]. For instance, proteins with KDEL-like retrieval signals rely on COPI-mediated pathways for their return to the ER. Disruption of this transport could affect the localization of key chaperones such as calnexin [24]. Notably, Arf-GAP has also been implicated in the pathogenicity of other fungi. For example, their deletion in Arthrobotrys oligospora compromises nematode capture [25]. Thus, the down-regulation of ArfGAP in EP713 may directly affect fungal pathogenicity by compromising COPI function, increasing ER stress, and interfering with secretion pathways essential for fungal virulence [26,27].

Taken together, our proteomic analysis reveals that the coordinated down-regulation of calnexin, COPI, and ArfGAP in EP713 may disrupts the balance between ER homeostasis and vesicle transport. Given that calnexin relies on COPI for proper localization, and COPI function is in turn regulated by ArfGAP, these changes may collectively impair ER function and retrograde transport, leading to elevated ER stress and compromised protein folding. Similar disruptions in ER-associated pathways have been linked to virulence attenuation in other fungi [28,29].

### 3.3. Possible Stress Responses Associated with Vesicle Trafficking Changes

Proteomics analysis revealed several up-regulated proteins involved in the formation of clathrin-coated vesicles (CCVs) in the trans-Golgi Network (TGN), particularly clathrin and dynamin. Clathrin forms a polygonal lattice to drive membrane budding [30], while dynamin assembles at the bud neck and severs the vesicle via GTP hydrolysis [31]. Vps10p, a key TGN sorting receptor, also increased expression, directing specific cargo into CCVs for delivery to endosomes and vacuoles [32]. This conserved trafficking pathway is essential for hydrolase transport [33]. The up-regulation of clathrin and dynamin in EP713 may reflect an increased reliance on CCV-mediated TGN–endosome–vacuole pathways, potentially contributing to the cellular capacity for handling misfolded or damaged proteins, a phenomenon reminiscent of vacuolar remodeling reported in heat-stressed yeast [34].

This apparent enhancement of a specific trafficking pathway may be part of a broader cellular response to proteotoxic stress, further supported by the significant up-regulation of Hsp70 in hypovirus-infected strain EP713. Hsp70 facilitates protein folding, prevents aggregation, and promotes degradation of misfolded proteins [35]. Its expression typically increases under stress, thereby enhancing cellular resilience [36]. Hsp70 (particularly Hsc70) also aids in clathrin-coated vesicle uncoating by binding the clathrin heavy chain and using ATP hydrolysis to disassemble the lattice [37]. This dual role positions Hsp70 at the intersection of proteostasis and vesicular trafficking, tightly linking these two stress-response pathways. Thus, the up-regulated Hsp70 may reflect an adaptive response to ER stress and support both protein quality control and vesicle recycling under hypovirus-induced dysfunction.

The combination of up-regulated TGN–endosome–vacuole trafficking components and Hsp70 may reflect a coordinated cellular response aimed at alleviating proteotoxic stress. Such a mechanism could serve as a compensatory quality control process by directing misfolded proteins toward vacuole degradation, thereby relieving ER burden [38]. However, remodeling of trafficking pathways may also have unintended consequences [39], potentially exploited by the hypovirus to suppress host defenses. Mechanisms with functional similarities have been reported in other systems, such as the bacterial effector RipD from Ralstonia solanacearum that targets VAMPs in plants, thereby weakening host defense [40], and the involvement of ER stress responses in regulating fungal virulence [41].

### 3.4. Altered Autophagy-Related Responses

Our proteomic analysis revealed a significant down-regulation of Atg8 in the hypovirus-infected strain EP713. This ubiquitin-like protein is essential for autophagy. It undergoes PE-conjugation to localize on the isolation membrane, promoting autophagosome expansion and cargo sequestration [42]. The decreased expression level of Atg8 likely contributed to impaired autophagic activity.

Autophagy is essential for fungal development, nutrient recycling, and stress adaptation, all of which are critical for pathogenesis [43]. In *C. parasitica*, cpatg8 is required for both fungal virulence and viral RNA accumulation, as its deletion leads to attenuated pathogenicity and reduced CHV1 replication. Previous studies have reported that cpatg8 is up-regulated following CHV1 infection at the transcriptome level [44]. Whereas our proteomic data revealed a decreased protein accumulation of Atg8. Discrepancies between mRNA and protein levels are common, with accumulating evidence indicating that mRNA levels only partially predict protein abundance. This is because post-transcriptional processes, such as translational efficiency and protein degradation, significantly influence final protein expression levels [45,46]. Similar patterns of transcriptional up-regulation with suppressed protein expression have been observed in mammalian antiviral responses [47]. Based on these findings, we hypothesize that CHV1 may transcriptionally activate cpatg8 to support its replication, while concurrently suppressing Atg8 protein accumulation through translational or post-translational regulation. This suppression may impair host autophagy, contributing to reduced fungal virulence, suggesting that CHV1 employs multiple strategies to manipulate key host genes.

Notably, the apparent suppression of Atg8-dependent autophagy in hypovirus-infected strain EP713 might be functionally compensated by enhanced vacuolar trafficking. Given our earlier observation of up-regulated TGN–endosome–vacuole transport and Hsp70 expression, the fungus was speculated to employ alternative quality-control pathways to mitigate proteotoxic stress. Rather than bulk autophagy, this vesicle related autophagy may facilitate selective degradation of damaged proteins or organelles [48].

### 3.5. Up-Regulation of t-SNARE Proteins and Potential Functional Shifts

Proteomic analysis of vesicle-associated proteins in EP713 revealed significant up-regulation of two t-SNARE proteins. t-SNARE is membrane-anchored proteins located at the target membrane, which pairs with vesicle-associated v-SNAREs to mediate specific membrane fusion [49]. The elevated expression of t-SNAREs in EP713 may indicate enhanced vesicle fusion events and possible reorganization of vesicle trafficking dynamics under hypoviral infection.

Up-regulated t-SNAREs can facilitate efficient fusion between endosomes and vacuoles or between the TGN and endosomes, potentially promoting hydrolase delivery or degradation of misfolded proteins [50]. Furthermore, the increased expression level of t-SNARE is likely to enhance retrograde transport from endosomes back to the TGN, which is essential for maintaining the recycling of sorting receptors such as Vps10p [51]. These changes may represent an adaptive strategy to fine-tune vesicle targeting and cargo specificity during hypovirus-infected stress.

### 3.6. Potential Impact of Down-Regulated Importin-β on Nuclear Transport and Pathogen Virulence

Importin-β is a key factor in protein transport into and out of the nucleus [52,53], and its dysfunction can lead to abnormal protein distribution, consequently affecting normal cellular physiology. Importin-β is involved not only in nuclear transport but also in cell cycle regulation [54] and organization of membrane systems [55]. Vesicular transport, a system highly dependent on membrane dynamics and the coordinated action of numerous proteins, can also be influenced by the expression level of importin-β [56].

In EP713, importin-β expression was notably down-regulated. Impaired nuclear transport may affect the expression and function of key virulence factors in the pathogen [57]. In addition, the disorganization of membrane systems could reduce the efficiency of the vesicular transport system, thereby affecting the secretion of effectors and metabolites. Such alterations are likely to modify the pathogen’s interaction with the host, potentially resulting in decreased virulence. [58]. Our results suggest that changes in importin-β expression may contribute to the attenuated fungal virulence observed in hypovirus-infected strains.

## 4. Conclusions

In conclusions, by integrating our current vesicle proteomics data with previously published transcriptomic and proteomic findings [13,14], we propose a working model in which CHV1 infection extensively remodels the host’s vesicular trafficking system, likely modulating cellular stress and defense pathways to support its life cycle.

A key aspect of this remodeling is the disruption of ER-Golgi retrograde trafficking. Our current proteomic data reveals a significant down-regulation of COPI, ArfGAP, and the ER chaperone calnexin. This finding is consistent with the induction of ER stress, as deficiencies in these individual components are linked to stress responses in *C. parasitica* [20] and other fungi [25,27]. Furthermore, our data suggests a complex modulation of autophagy. While previous transcriptomic work showed an upregulation of the cpatg8 gene [44], we observed a reduction in Atg8 protein abundance, pointing towards post-transcriptional regulation as a potential viral strategy to dampen autophagic activity.

Concurrently, the host appears to activate compensatory pathways. The up-regulation of clathrin and Vps10p in our dataset suggests an enhanced capacity for the TGN-to-vacuole degradative pathway, likely to clear misfolded or viral proteins. This aligns with broad transcriptional changes in stress-response processes reported by Chun et al. [14] and our own previous findings of increased vesicle-associated stress proteins like Hsp88. This large-scale reprogramming of secretion is further evidenced by the up-regulation of t-SNARE proteins in our current data. This finding is consistent with our earlier work, which showed not only a selective reduction in metabolic enzymes within vesicles but also an enrichment of vesicle-associated components such as V-ATPase subunits, ABC transporters, and Hsp88, alongside the presence of viral proteins like p48.

**Figure 5:**
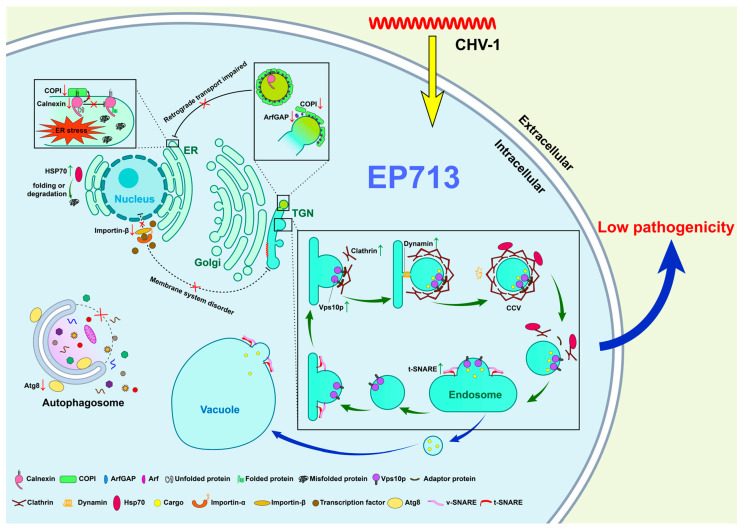
CHV1-induced alterations in key trafficking components, including calnexin, COPI, ArfGAP, importin-β, Atg8, HSP70, clathrin, dynamin, and SNAREs, reconfigure the vesicular network.

Collectively, these convergent lines of evidence from proteomics and transcriptomics paint a cohesive picture of viral takeover (Figure 5). CHV1 appears to suppress core host pathways like retrograde transport while enhancing specific degradative routes, likely to manage viral protein load and redirect host resources. However, as this model is based on correlational data, direct functional tests, such as the genetic perturbation of these key trafficking factors, are required to validate causality.

## Figures and Tables

**Table 1:** Information on proteins encoded by hypovirus.

Accession	Description
Q04350	ORFB polyprotein OS = Cryphonectria hypovirus 1 (strain EP713) OX = 12478 PE = 1 SV = 1 − [POLB_CHPVE]
A0A2H4UK75	RNA-directed RNA polymerase OS = Cryphonectria hypovirus 1 OX = 40281 PE = 4 SV = 1 − [A0A2H4UK75_9VIRU]
A0A2H4UK88	RNA-directed RNA polymerase OS = Cryphonectria hypovirus 1 OX = 40281 PE = 4 SV = 1 − [A0A2H4UK88_9VIRU]
A0A2H4UK77	RNA-directed RNA polymerase OS = Cryphonectria hypovirus 1 OX = 40281 PE = 4 SV = 1 − [A0A2H4UK77_9VIRU]
A0A2H4UK87	RNA-directed RNA polymerase OS = Cryphonectria hypovirus 1 OX = 40281 PE = 4 SV = 1 − [A0A2H4UK87_9VIRU]
A0A2H4UK85	RNA-directed RNA polymerase OS = Cryphonectria hypovirus 1 OX = 40281 PE = 4 SV = 1 − [A0A2H4UK85_9VIRU]
P10941	Polyprotein p69 (precursor to p29 and p40) OS = Cryphonectria hypovirus 1 (strain EP713) OX = 12478 PE = 1 SV = 2 − [POLA_CHPVE]
A0A2H4UK83	ORF A OS = Cryphonectria hypovirus 1 OX = 40281 PE = 4 SV = 1 − [A0A2H4UK83_9VIRU]
E2GDE0	Polyprotein OS = Cryphonectria hypovirus 1 OX = 40281 PE = 4 SV = 1 − [E2GDE0_9VIRU]
A0A2H4UK61	ORF A OS = Cryphonectria hypovirus 1 OX = 40281 PE = 4 SV = 1 − [A0A2H4UK61_9VIRU]
W8EC18	OrfA polyprotein (Fragment) OS = Cryphonectria hypovirus 1 OX = 40281 GN = OrfA PE = 4 SV = 1 − [W8EC18_9VIRU]
A0A2H4UK73	ORF A OS = Cryphonectria hypovirus 1 OX = 40281 PE = 4 SV = 1 − [A0A2H4UK73_9VIRU]
Q9YME5	Peptidase C7 domain-containing protein OS = Cryphonectria parasitica hypovirulence associated virus OX = 83190 PE = 4 SV = 1 − [Q9YME5_9VIRU]
Q7TDB9	OrfA (Fragment) OS = Cryphonectria hypovirus 1 OX = 40281 PE = 4 SV = 1 − [Q7TDB9_9VIRU]
Q7T598	ORF B (Fragment) OS = Cryphonectria hypovirus 1 OX = 40281 PE = 4 SV = 1 − [Q7T598_9VIRU]
Q7T594	ORF B (Fragment) OS = Cryphonectria hypovirus 1 OX = 40281 PE = 4 SV = 1 − [Q7T594_9VIRU]
E2GDC9	p48 (Fragment) OS = Cryphonectria hypovirus 1 OX = 40281 PE = 4 SV = 1 − [E2GDC9_9VIRU]
A0A7T0Q710	ORF-A polyprotein (Fragment) OS = Cryphonectria hypovirus 1 OX = 40281 PE = 4 SV = 1 − [A0A7T0Q710_9VIRU]

## Data Availability

The original contributions presented in the study are included in the article/Supplementary Material, further inquiries of the raw data of fungal vesicle proteome can be directed to the corresponding authors.

## Supplementary Materials

The following supporting information can be downloaded at https://www.mdpi.com/article/10.3390/biology14091123/s1. Table S1: List of identified fungal proteins in vesicles of *C. parasitica*; Table S2: Relative quantitative analysis of PRM; Table S3: List of differentially expressed proteins upon hypovirus infection; Table S4: Detailed KEGG pathway enrichment results including raw and adjusted *p*-values. Figure S1: Western blot analysis of fungal vesicle.
