# Supplementary figures and images for: Evaluation of Hypovirus Infection on the Vesicular Protein Expression Pattern of Cryphonectria parasitica by TMT-Based Proteomics Analysis
Source: Biology (Basel). 2025 Aug 25;14(9):1123. doi: 10.3390/biology14091123 (PMC12467672; doi:10.3390/biology14091123)

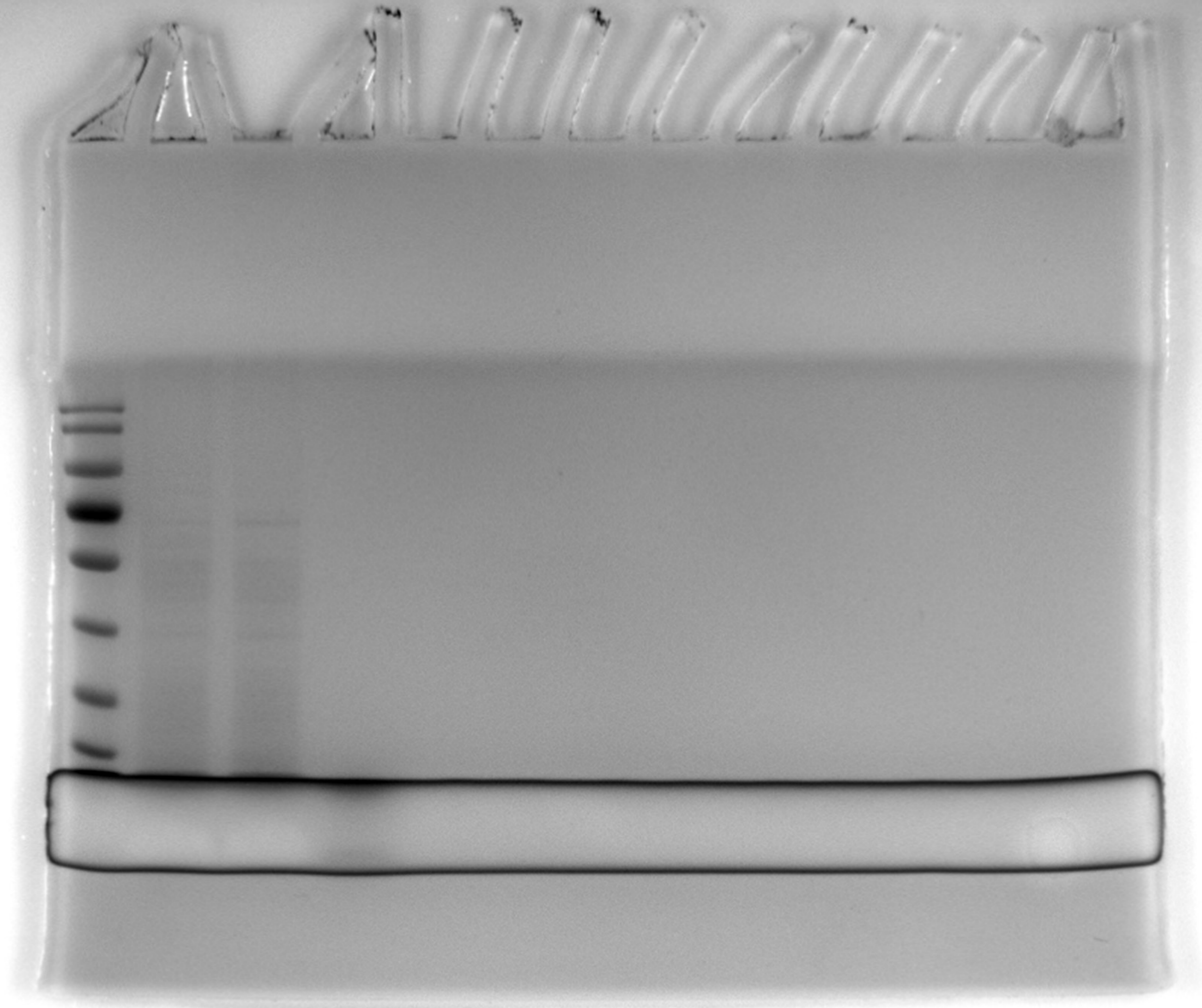

Supplement: Supplementary file 1 [file biology-14-01123-s001.zip › Figure S1.pdf]
